# Adverse drug events in Chinese elder inpatients: a retrospective review for evaluating the efficiency of the Global Trigger Tool

**DOI:** 10.3389/fmed.2023.1232334

**Published:** 2023-09-28

**Authors:** Nan Yu, Liuyun Wu, Qinan Yin, Shan Du, Xinxia Liu, Shan Wu, Rongsheng Tong, Junfeng Yan, Yuan Bian

**Affiliations:** ^1^Department of Pharmacy, Sichuan Academy of Medical Sciences and Sichuan Provincial People’s Hospital, School of Medicine, University of Electronic Science and Technology of China, Chengdu, China; ^2^Chengdu First People’s Hospital, Chengdu, China; ^3^Personalized Drug Therapy Key Laboratory of Sichuan Province, School of Medicine, University of Electronic Science and Technology of China, Chengdu, China; ^4^Maternal and Child Health Hospital of Shuangliu District, Chengdu, China

**Keywords:** adverse drug events, adverse drug reaction, Global Trigger Tool, retrospective study, medication management

## Abstract

**Background:**

Elderly patients frequently experience a high incidence of adverse drug events (ADEs) due to the coexistence of multiple diseases, the combination of various medications, poor medication compliance, and other factors. Global Trigger Tool (GTT) is a new method for identifying ADEs, introducing the concept of a trigger, that is, clues including abnormal laboratory values, reversal drugs, and clinical symptoms that may suggest ADEs, and specifically locating information related to ADEs in the medical record to identify ADEs. The aim of this study was to establish a GTT-based trigger tool for adverse medication events in elderly patients and to investigate the risk variables associated with such events.

**Methods:**

The triggers were identified by reviewing the frequency of ADEs in elderly patients in Sichuan, China, retrieving relevant literature, and consulting experts. A retrospective analysis was carried out to identify adverse medication occurrences among 480 elderly inpatients in Sichuan People’s Hospital.

**Results:**

A total of 56 ADEs were detected in 51 patients (10.62%), 13.04 per 1,000 patient days, and 11.67 per 100 admissions. The overall positive predictive value (PPV) of the triggers was 23.84, and 94.64% of ADEs caused temporary injury. Gastrointestinal system injury (27.87%) and metabolic and nutritional disorders (24.53%) were the primary organ-systems affected by ADEs. The majority of ADEs were caused by drugs used to treat cardiovascular diseases. 71.43% of ADE occurred within 2 days of administration and the risk factor analysis of ADE revealed that the number of medicines had a significant correlation.

**Conclusion:**

This study demonstrated GTT’s value as a tool for ADEs detection in elderly inpatients in China. It enhances the level of medication management and comprehensively reflects the situation of ADE of the elderly.

## 1. Introduction

Since the late 1990s, the aging of Chinese society has entered a period of sustainable acceleration. In 2018, the population aged 65 and over in China reached 166 million, accounting for 11.9% of the total population (1). In 2021, senior patients (≥65) accounted for 26% according to the report data from the National Drug Reaction Monitoring Center of China, which indicated an upward trend since 2009 (2). There is a significant prevalence of adverse drug events (ADEs) among elderly patients with degenerative changes in the structure and physiological activities of the organs, especially those with chronic conditions, due to multi-drug combination therapy and poor medication compliance (3, 4). 10%–30% of elderly patients are admitted to hospital due to ADEs (5), 4.93% were emergency admissions, and more than 80% of emergency ADE-related hospitalizations were patients over 60 years old (6). The incidence of ADEs in elderly patients in the same period accounted for 53.1% of the total ADEs (7).

From 1999 to 2020, China’s National Adverse Drug Reaction Monitoring Network received a total of 16.87 million copies of Adverse Drug Reaction (ADR)/ADE reports, compared with 1.676 million in 2020, of which 506,000 were new and severe ADEs (2). ADE-related deaths and trends have increased, especially in the elderly population (8), and are associated with longer hospital stays, a tripling risk of death, and increased costs (9). ADE has grown to be a serious public health issue that jeopardizes the security of pharmacological therapy globally and is one of the leading causes of iatrogenic injuries (10). The traditional way of monitoring is spontaneous reporting, however, this unreflective, solitary manner with under- and misreporting cannot accurately reflect the occurrence of ADEs. The Global Trigger Tool (GTT), developed by the Institute for Healthcare Improvement (IHI) in 2003, seeks to identify “triggers” in the review process and specifically locates ADE-related information in medical records, so as to provide clues of analysis and identification (11). Triggers are divided into six modules in the GTT white paper, including medication, care, emergency department, surgery, intensive care, and perinatal. There are 13 triggers in the drug module, such as laboratory, antidotes, clinical, etc. GTT has currently been widely studied and applied. Studies in the U.S., Sweden, Turkey, and South Korea have confirmed that GTT has good effectiveness and practicability, with 19–50 times higher efficiency in detection compared to conventional methods (12–15). There was limited research on elderly individuals, with the majority of GTT subjects being general inpatients and pediatric populations. Recent studies on elderly individuals in Australia (16) and Spain (17–19) have demonstrated that GTT is practical and reasonable. However, previous studies have suggested that the trigger should be modified and improved in accordance with clinical medication use and demographic characteristics when GTT is applied in various geographic locations and research populations. In addition, we know very little about the application of GTT and the occurrence of ADEs in Chinese elderly inpatients. Therefore, our objectives were to determine the relationship between triggers and ADEs, to improve the functionality of the trigger tool, and to explain the characteristics and incidence of the ADEs detected by using this tool in older.

## 2. Methods

### 2.1. Study resources

Domestic and foreign literature reported that the incidence of ADE in elderly hospitalized patients was about 10% (*P*), and the sample size *N* = 384 was calculated by selecting a 95% confidence level (i.e., the statistic *Z* was 1.96) and a 3% sampling error (*δ*) according to the formula *N* = *Z*^2^ × *P* × (1 − *P*)/*δ*^2^. To appropriately expand, a total of 480 medical records of patients who were discharged from the hospital’s geriatrics department between January 1 and December 31, 2021, were chosen based on the informed and voluntary principle and the following screening criteria, and 40 records were randomly chosen each month with the PASS clinical system of Medicom Software. The included patients were older than 65 years old with the length of stay exceeded 1 day. Elderly patients with malignant tumors, organ transplants, palliative care, and transfers to ICU were excluded because they were prone to experience ADEs after using drugs due to their special physiological conditions and it was difficult to determine whether these reactions were brought on by medicines or the diseases they were suffering from.

### 2.2. Triggers

The trigger items in this study were established based on the triggers recommended in the IHI white paper of GTT, previous studies on trigger tools, and reports from the Sichuan provincial center for monitoring adverse drug reactions. The trigger list was ultimately created by a conversation among the specialists. There were 36 triggers in the list, including 17 laboratory indexes, 10 treatments, 8 clinical symptoms, and 1 intervention measure. The triggers of this study were primarily intended for elderly patients without cancer.

### 2.3. Records review

Two primary reviewers (pharmacists) and two senior reviewers (physician and pharmacist) make up the record review team, and the characteristics of each reviewer are detailed in Supplementary Table S1. First of all, primary reviewers reviewed the records separately in accordance with the standard procedure of the triggers. The following medical documents were examined: admission record, medication administration record (long-term and temporary), laboratory results, nursing notes, patient consultation and emergency records, etc. (11). The review of each record took 30 min due to the numerous complications and drug use in the elderly patients. During the review, information on patients was noted including their basic data (sex, age), length of stay, history of drug allergy, number of medications, trigger-specific information, occurrence time, and so on. The senior reviewers then answered any queries that had been raised by the two primary reviewers during the review and verified the outcomes of the ADE and the severity rating findings. The final review results were discussed in the research group meeting.

ADEs in this study occurred in the cases with qualified pharmacological therapy, meaning that the ones due to the quality of the drug were excluded. The relevance was evaluated with the Naranjo scale (20, 21), and was divided into definite, probable, possible, or doubtful. The definite and probable were considered in this investigation.

The severity of ADE is evaluated by the Common Terminology Criteria for Adverse Events (CTCAE, Version 5.0) (22). It contained mild symptoms and intervention not indicated (Grade 1), minimal, local, or noninvasive intervention indicated (Grade 2). Severe or medically significant but not immediately life-threatening; hospitalization or prolongation of hospitalization (Grade 3), Life-threatening consequences; urgent intervention indicated (Grade 4) and death (Grade 5).

### 2.4. Statistical analysis

Data were analyzed by Microsoft Excel 2016 and SPSS21.0 software. The rank sum test and chi-square test were used to compare the quantitative and qualitative data, and the binary logistic regression method was used to analyze the influencing factors of ADE in elderly patients. We calculated ADEs per 1,000 patient days, ADEs per 100 admissions, and the occurrence rate of ADE in hospitalized patients (11), in which the ADEs per 1,000 patient days was an index to track the occurrence of ADE over time. The evaluation index of the trigger was positive predictive value (PPV). An ADE may be identified by more than one trigger. Finally, according to the results, the trigger is corrected and improved.

## 3. Results

### 3.1. Patients characteristics

A total of 480 cases were randomly selected. The mean age was 72.61 years (65–91 years), of which more than half were male (54.38%). The mean length of stay was 8.87 ± 4.61 days (1–27 days). The average number of medical diagnoses was 5.31 ± 2.79 (1–19), and the average number of medications per patient was 17.93 ± 6.42 (5–40). 30.83% of the patients used antibacterial during hospitalization, and the main reason for use was pulmonary infection. The average duration of antibacterial drugs was 52.66 ± 102.31 h (3–528 h). 14.38% of the patients had a history of drug allergy, mainly to penicillin and sulfonamides. There were only significant differences in the number of medications between patients with and with no ADEs (*p* < 0.01) (Table 1).

**Table 1:** Patient characteristics.

Characteristics	Total (*n* = 480)	Patient with ADEs (*n* = 51)	Patients without ADEs (*n* = 429)	*p*
Age	72.61 ± 5.94	72.18 ± 5.47	72.66 ± 5.98	0.657
Female ratio (%)	45.62% (219/480)	43.14% (22/51)	45.92% (197/429)	0.768
Length of stay (days)	8.95 ± 4.57	9.59 ± 4.14	8.87 ± 4.61	0.196
Medical diagnoses	5.38 ± 2.80	6.04 ± 2.82	5.31 ± 2.79	0.062
Medications per patient^a^	18.26 ± 6.42	21.04 ± 5.68	17.93 ± 6.42	0.001
Antibacterial use				0.749
Yes	148 (30.83%)	17 (33.33%)	131 (30.54%)	
No	332 (69.17%)	29 (66.67%)	298 (69.46%)	
Length of antibacterial use (h)	53.42 ± 103.24	59.76 ± 110.58	52.66 ± 102.31	0.667
Drug allergy history				0.835
Yes	69 (14.38%)	8 (15.69%)	61 (14.22%)	
No	411 (85.62%)	43 (84.31%)	368 (85.78%)	

a Indicates that the *p*-value used for statistical significance was *p* < 0.05.

### 3.2. Triggers

A total of 281 positive triggers were identified from the 480 cases, involving 232 patients (48.33%). Among the 36 triggers, 31 (86.11%) were triggered positively, and 17 were associated with ADEs. The overall PPV of the triggers was 23.84%, the frequency of positive triggers, detected ADEs, and PPV for each trigger are shown in Table 2.

**Table 2:** The triggers and PPV.

Modules	No.	Triggers	Interpretation	Positive triggers	ADEs	PPV (%)
Laboratory index	L1	K^+^ <3.0 mmol/L (18, 23)	Hypokalemic drugs used	6	1	16.67
	L2	K^+^ >6 mmol/L; K^+^ >5.5 mmol/L and eGFR <50 mL/min (23)	Hyperkalemic drugs used	3	0	0
	L3	Na^+^ <130 mmol/L (18, 24)	Hyponatremic drugs used	11	0	0
	L4	No diabetes: serum glucose <2.8 mmol/L; Diabetes: serum glucose <3.9 mmol/L (18, 25, 26)	Hypoglycemic drugs used	13	2	15.38
	L5	No diabetes: fasting blood glucose ≥7.0 mmol/L, postprandial blood glucose ≥7.8 mmol/L; Diabetes: serum glucose levels are higher than in the past (note: elevated blood sugar is not associated with the primary disease, and stress hyperglycemia is excluded) (18, 26)	Hyperglycemia drugs used or hypoglycemic drugs used inappropriately	4	1	25.00
	L6	ALT and ALP ≥2 × ULN (27) (exclude patients who use parenteral nutrition drugs and have gallbladder, pancreatic or liver disease)	Drugs induced liver damage	12	3	25.00
	L7	Creatinine >2 times base value^a^ GFR decreased by 25% and/or urine volume <0.5 mL/(kg·h), duration >12 h (28, 29)	Drugs induced kidney injury	1	0	0
	L8	PT >12.1 s; APTT >36.5 s; INR >3.5^b^	Drugs induced clotting dysfunction	3	1	33.33
	L9	Platelets count <50 × 109/L (18, 23, 24)	Drugs induced thrombocytopenia	4	0	0
	L10	(1) TSH ≥5.0 mIU/L; (2) TSH <0.3 mIU/L (excluding patients with thyroid dysfunction) (24, 30)	Drugs induced thyroid dysfunction	9	0	0
	L11	Leukocyte count <3 × 109/L (18, 23, 25)	Drugs induced leukopenia	4	0	0
	L12	(1) Different degrees of myalgia/myasthenia/myositis (with or without elevated CK levels); (2) Creatine kinase >10 times of the upper limit of normal value; (3) Abnormal urine examination (dark brown, myoglobin urine); (4) With or without renal function damage oliguria or serum creatine at least increase 0.5 mg/dL (44 μmol/L) (no primary muscle disease, rhabdomyolysis if the conditions are met; if not fully satisfied, associated myopathy) (31)	Drugs induced myopathy	3	0	0
	L13	Hemoglobin >120 g/L and the patient has chronic renal failure (24)	Administrated recombinant human erythropoietin	1	0	0
	L14	Electrocardiographic abnormality (32)	Drug-induced arrhythmias	2	2	100.00
	L15	Uric acid >428 μmol/L (33) (the patient had no history of hyperuricemia and gout)	Drugs induced increasing uric acid	73	8	10.96
	L16	Systolic pressure <90 mmHg (11, 25, 34)	Drugs induced lower blood pressure	4	3	75.00
	L17	Theophylline >20 mg/L; Digoxin >2 ng/mL; Peak concentration of vancomycin >20 mg/L; Gentamicin >10 mg/L; Carbamazepine >13 mg/L (18, 23–25, 35, 36)	Theophylline/digoxin/vancomycin/gentamicin/carbamazepine overdose	1	1	100.00
Treatments	T1	Vitamin K administration (11, 23)	Vitamin K antagonists used	0	0	—
	T2	Protamine use (23)	Heparin overdose	0	0	—
	T3	(1) Serum glucose >13.9 mmoL/L:500 mL0.9%NaCl^+^ short-acting insulin + KCl/NaHCO_3_ solution was used; (2) Serum glucose <13.9 mmoL/L: 500 ml 5% glucose solution (sodium chloride glucose injection) + short-acting insulin + KCl/NaHCO_3_ intravenous drip solution (26)	After drug-induced acidosis	7	0	0
	T4	Antihistamine drugs/calcium gluconate or combined use of glucocorticoid/epinephrine (37)	After drug allergy	8	7	87.50
	T5	Intravenous infusion of 50% glucose injection (26)	After drug induced hypoglycemia	4	0	0
	T6	Intestinal live bacteria/oral vancomycin/oral metronidazole use (patients have a long history of using antibiotics/PPIs) (23)	Diarrhea caused by antibiotics or PPIs	2	0	0
	T7	Hepatoprotective drug use (37)	Drug-induced liver injury	29	2	6.90
	T8	Flumazenil use (23, 25)	Relief of severe hypotension and prolonged sedation caused by an overdose of benzodiazepines	0	0	—
	T9	Nystatin and sodium bicarbonate use (23)	Long-term antibiotic/hormone/immunosuppressive associated Candida infection (thrush)	0	0	—
	T10	Use phenytoin/atropine/lidocaine (23)	Relief of ventricular tachycardia, bradycardia, supraventricular and ventricular tachycardia caused by digoxin poisoning	1	1	100.00
Symptoms	C1	Rash (11, 25)	Drugs induced allergic reactions to the skin	13	8	61.54
	C2	Over sedation/falls (11, 25, 34)	Used antihypertensive drugs, sedative hypnotic drugs, anti-Parkinson’s, hypoglycemic drugs	1	0	0
	C3	Delirium (29)	Drugs induced neurological disorders	1	1	100.00
	C4	Epilepsy	Drugs induced epileptic seizures	1	0	0
	C5	Cognitive disorder (patients with no dementia, Parkinson’s disease or other degenerative cognitive diseases)	Drugs that affected cognitive function	0	0	—
	C6	Edema	Drugs induced edema	15	0	0
	C7	Hemorrhage	(1) Used platelet inhibitors, heparin, low molecular weight heparin, vitamin K antagonists, direct oral anticoagulants or anticoagulants in combination with selective 5-HT reuptake inhibitors; (2) Patients with a history of peptic ulcer took NSAIDs/corticosteroid drugs, but did not regularly take PPI preparations	13	3	23.08
	C8	Dry cough (the patient did not cough or did not cough often before taking the medication)	ACEI drugs used	3	2	66.67
Intervention measure	I1	Abrupt medication stops (11, 18, 23, 25, 35)	Medication may be discontinued due to the presence of ADE requiring adjustment of medication	29	21	72.41
	Total			281	67	23.84

ADE, adverse drug events; PPV, positive predictive value; eGFR, glomerular filtration rate; ALT, alanine aminotransferase; ALP, alkaline phosphatase; ULN, upper limits of normal; PT, prothrombin time; APTT, activated partial thromboplastin time; INR, international normalized ratio; TSH, thyroid-stimulating hormone; CK, creatine kinase; PPIs, proton pump inhibitors.

a (Determination of the base value: (1) if the value of creatine anhydride was not measured at admission, it was assumed that the previous renal function of the patient was normal; (2) the minimum Scr during hospitalization was used as the basic value. The lowest of the above two).

b Trigger for adjustment according to specific drug and actual application in our hospital.

### 3.3. ADE characteristics

**Figure 1:**
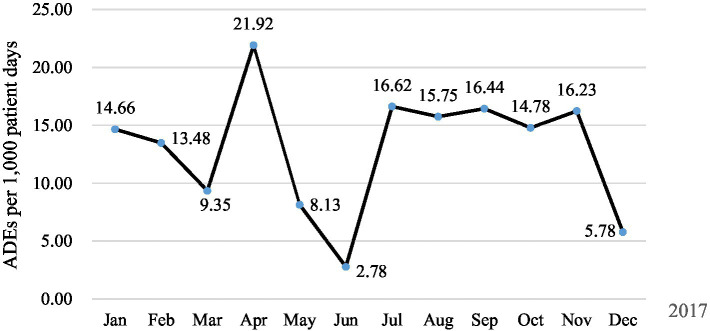
ADEs per 1,000 patient days in 2021.

**Figure 2:**
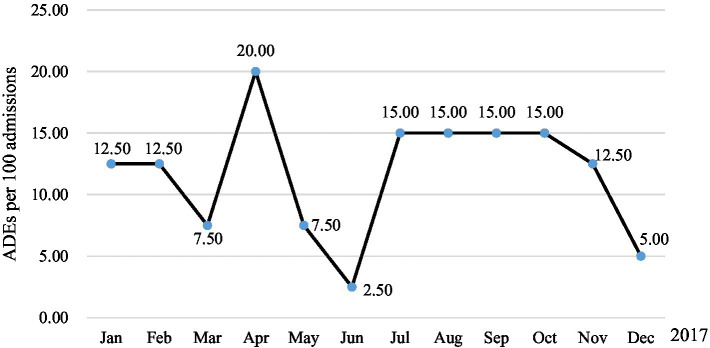
ADEs per 100 admissions in 2021.

ADE association evaluation results showed that a total of 56 ADEs were detected in 51 patients (10.62%), whereas, 4 ADEs were not detected by triggers. Of these, 47 (9.79%), 3 (0.62%), and 1 (0.21%) patients had one, two, and three ADEs, respectively. Two ADEs were medication errors, including prescribing errors drug interactions and medication compliance errors (irregular use of hypoglycemic drugs led to elevated serum glucose). Regarding occurrence rates, 94.64% (53/56) occurred during hospitalization, and 53.57% (30/56) of ADE occurred within 1 day after administration (Table 3). One patient was admitted to hospital with ADE (Poisoning by digoxin). The overall incidence of ADEs was 10.62% (51/480), 13.04 per 1,000 patient days (Figure 1), 11.67 per 100 admissions (Figure 2).

**Table 3:** ADEs occurrence time.

Occurrence time	No.	Total No. (%)
≤5 h	7	12.50
5 h–1 day	23	41.07
1–2 days	12	21.43
2–4 days	9	16.07
4–8 days	2	3.57
Prior to admission	3	5.36
Total	56	100

Reagrding classification, 11 ADEs (19.64%) were classified into Grade 1 according to the severity of CTCAE, 42 (75.00%) were Grade 2, and 3 (5.36%) were Grade 3. The main manifestation of ADE was gastrointestinal system damage (27.87%) (Table 4) and most ADEs were mainly caused by drugs for treating cardiovascular disease, such as digoxin, amiodarone, aspirin, furosemide, etc.

**Table 4:** ADEs involved system-organ classification.

System/organ	Clinical symptoms	No.	Total No. (%)
Gastro-intestinal system disorders	Nausea/vomiting	8	27.87
	Diarrhoea/increased stool frequency/abdominal pain	6	
	Gastrointestinal haemorrhage/faeces discoloured	3	
Metabolic and nutritional disorders		8	24.59
	Hypokalaemia	4	
	Hypoglycaemia	2	
	Hyperglycaemia	1	
Skin and appendages disorders	Rash	5	14.75
	Dermatitis/pruritus	3	
	Erythema exudativum	1	
Cardiovascular disorders, general	Hypotension	3	6.56
	Electrocardiograph abnormal	1	
Liver and biliary system disorders	Hepatic function abnormal	3	4.92
Central and peripheral nervous system disorders	Dizziness/headache	3	4.92
Heart rate and rhythm disorders	Palpitation/QT increased	2	3.28
Platelet, bleeding and clotting disorders	Epistaxis/coagulation time increased	2	3.28
Autonomic nervous system disorders	Sweating increased	2	3.28
Respiratory system disorders	Dry cough	2	3.28
Body as a whole-general disorders	General malaise	1	1.64
Vision disorders	Vision abnormal	1	1.64
Total		61	100

Because the one ADE may have multiple clinical manifestations at the same time, the total number of cases in the table is larger than that number of ADE.

### 3.4. Risk factors associated with the occurrence of ADEs

Previous studies have shown that the number of drugs used, the length of stay, the number of medical diagnoses and the use of antibacterial are important factors affecting the incidence of ADE in elderly patients (19, 38, 39). The occurrence of ADE was used as dependent variable and the above factors as independent variables for regression analysis, the results showed that only the number of drugs used was statistically significant (Table 5).

**Table 5:** Logistic regression analysis of influencing factors of ade in elderly patients.

Variables	*β*	SE	Odds ratio	*p*	95% CI
Length of stay (days)	−0.032	0.043	0.968	0.460	0.889–1.055
Medical diagnoses	0.069	0.060	1.072	0.247	0.953–1.204
Medications per patient^a^	0.087	0.028	1.091	0.002	1.032–1.153
Antibacterial use	−0.111	0.321	0.895	0.730	0.477–1.680
Length of Antibacterial use	−0.001	0.002	0.999	0.392	0.995–1.002

a Indicates that the *p*-value used for statistical significance was *p* < 0.05.

## 4. Discussion

The incidence of ADE in elderly inpatients in this study was 10.62%, which was consistent with the incidence in Canada, Japan, and Malaysia (6.3%–15.8%) (40–42). It was significantly higher than the voluntary reporting rate of 2.34% (79/3341) in our hospital during the same period, but lower than 24.7% of Toscano et al. (19), which may be related to the criteria of ADE and the scope of the study population. In the study, the incidence of ADE was significantly lower in May and June, which was possibly due to sampling errors. The results of the severity grading of 56 ADE patients according to the CTCAE standard showed that 94.64% of ADE caused temporary injury and could be cured or improved without treatment or given certain interventions. The detected ADE is mainly caused by cardiovascular drugs, which may be due to the high prevalence of cardiovascular diseases in the elderly population. The statistics of heart disease and stroke data released by the American Heart Association (AHA) in 2017 show that, cardiovascular disease is the leading cause of death in the world (43). Cardiovascular drugs have become commonly used in drug therapy in elderly patients, led to the prevalence of ADE. ADE mainly caused by cardiovascular drugs include hypotension, gastrointestinal bleeding, abnormal electrocardiogram, cough, etc. It is worth noting that 9 patients had delayed adverse drug events due to the use of iodixanol, 8 patients had hypersensitive reactions, and 7 patients were male patients, which was consistent with the conclusion that male was independent risk factors for iodixanol delayed ADR (44). As a commonly used contrast agent in clinics, iodixanol is most commonly used in cardio-cerebral vascular examination. Before examination, patients, especially male patients, should be asked in detail if they have a history of contrast agent allergy in order to avoid related adverse drug events. In addition, pay attention to drug interactions when other drugs are combined with drugs of the cardiovascular system. For example, we found that one patient was administered cefoperazone/tazobactam after warfarin use in the medical record review. The patient’s INR increased from 1.81 to 4.43, but no bleeding symptoms occurred, so only warfarin and cefoperazone/tazobactam were discontinued and there was no need to use vitamin K for rescue. Clinical pharmacists should conduct medication review in time after medication orders are issued by physicians to avoid adverse drug events in patients due to prescription errors.

Analysis of the influencing factors of ADE occurrence in elderly patients in this study showed that the number of drugs only had a significant impact on the occurrence of ADE, which was inconsistent with risk factors in other studies (such as age, length of stay in hospital, number of total doses of drugs, duration of use of antimicrobial agents, severity of disease, etc. (19, 39)). The reasons may mainly include the following two points. First, the sample size included in this study is limited. If the sample size is appropriately expanded, the risk factors mentioned above may show a significant correlation. Secondly, this study excluded elderly patients with malignant tumors, organ transplantation, palliative treatment, and transferred to ICU. ADE is very common in such patients who take anti-tumor drugs, immunosuppressive drugs, anti-infective drugs, etc. If such patients are included, the detection rate of ADE will also increase.

The overall PPV of the trigger was 23.84%, higher than the PPV (19.50%) of the older patients triggers list reported by Toscano et al. (19). The reason may be that more restrictions were set on the trigger conditions in this study which excluded the effects of related diseases. Another 15 triggers did not identify any ADE. For example, edema was found 15 times in the record review and no ADE was detected. The clinical manifestations of edema may occur in patients with cardiovascular disease or diabetes, so it’s easy to find. Depending on the results, hyponatremia, drug-induced thyroid dysfunction, and Hepatoprotective drug use were considered to be eliminated. Blood sodium level is affected by a variety of factors, such as heart, kidney, thyroid function, etc., making it difficult to determine whether drugs cause abnormal blood sodium levels. Generally, drug-induced thyroid dysfunction can be found at least 1 week after administration (45). The degenerative changes in body function would be undergone by the elderly patient. During the review, some patients were found to have subclinical hypothyroidism upon admission.

There were 5 items that were not triggered in this medical record review. Because the intervention measures of reducing the dose of suspected drugs were conducted after patients had nausea, headache, and an increase in stool frequency, the adverse reaction symptoms were gradually relieved and disappeared. Consider changing the trigger “abrupt medication stop to “abrupt medication stops or reduction of the drug dose.” It is necessary to exclude a reasonable dose adjustment at the time of concomitant medication in the course of review. In addition, hypokalemia (plasma potassium decreased from 4.56 mmol/L to 3.34 mmol/L after using furosemide) occurred in one patient. Although the index of hypokalemic <3.0 mmol/L was considered to be changed to <3.5 mmol/L, this may cause the increase of triggering false positive rate. All of this suggests that improving triggers needs to be based on clinical practice.

This study showed that the GTT was a useful tool for the detection of ADEs in elderly inpatients in China. The limitations of this study are mainly that the sample size is not large enough and the scope of research objects is limited. In the later stage, the trigger can be modified, corrected, and improved by expanding the sample size and population scope, preferably with a review of each case of an elderly hospitalized patient to be more convincing. To our knowledge, this was the first study to establish a trigger tool for monitoring adverse drug events in elderly hospitalized patients in China. More than one in 10 elderly hospitalized patients had adverse drug events, and most of them were temporary harm. GTT can provide assistance for the monitoring of ADE for the elderly in local medical institutions, and help to comprehensively reflect the situation of ADE for the elderly, so as to ensure the safety of medication and improve the level of drug management.

## Data availability statement

The original contributions presented in the study are included in the article/Supplementary material, further inquiries can be directed to the corresponding authors.

## Ethics statement

The experimental protocol was established, according to the ethical guidelines of the Helsinki Declaration and was approved by the Human Ethics Committee of Sichuan Academy of Medical Sciences & Sichuan Provincial People’s Hospital. Written informed consent was obtained from individual or guardian participants.

## Author contributions

NY, LW, QY, SD, XL, SW, RT, JY, and YB contributed to the study conception, design, and commented on previous versions of the manuscript. Material preparation, data collection and analysis were performed by NY, QY, and LW. The first draft of the manuscript was written by NY and QY. All authors contributed to the article and approved the submitted version.

## Glossary

ADE: Adverse drug events
ALP: Alkaline phosphatase
ALT: Alanine aminotransferase
APTT: Activated partial thromboplastin time
CK: Creatine kinase
CTCAE: Common Terminology Criteria for Adverse Events
eGFR: Glomerular filtration rate
GTT: Global Trigger Tool
IHI: Institute for Healthcare Improvement
INR: International normalized ratio
PPIs: Proton pump inhibitors
PPV: Positive predictive value
PT: Prothrombin time
TSH: Thyroid-stimulating hormone
ULN: Upper limits of normal
