# Supplementary material for: Adverse drug events in Chinese elder inpatients: a retrospective review for evaluating the efficiency of the Global Trigger Tool
Source: Front Med (Lausanne). 2023 Sep 28;10:1232334. doi: 10.3389/fmed.2023.1232334 (PMC10568622; doi:10.3389/fmed.2023.1232334)
Supplement: Supplementary file 1 [file Table_1.pdf]

Supplementary table 1 the characteristics of each reviewer

Rating	No.	Age (years)	Genger	Specialty	Experience (years)
Primary	1	27	Female	Pharmacy	3
Primary	2	28	Female	Clinical Pharmacy	3
Senior	3	31	Female	Clinical Pharmacy	3
Senior	4	35	Male	Clinical Pharmacy	6
